# Transposon identification using profile HMMs

**DOI:** 10.1186/1471-2164-11-S1-S10

**Published:** 2010-02-10

**Authors:** Paul T Edlefsen, Jun S Liu

**Affiliations:** 1 Department of Statistics, Harvard University, One Oxford Street, Cambridge, MA, USA

## Abstract

**Background:**

Transposons are "jumping genes" that account for large quantities of repetitive content in genomes. They are known to affect transcriptional regulation in several different ways, and are implicated in many human diseases. Transposons are related to microRNAs and viruses, and many genes, pseudogenes, and gene promoters are derived from transposons or have origins in transposon-induced duplication. Modeling transposon-derived genomic content is difficult because they are poorly conserved. Profile hidden Markov models (profile HMMs), widely used for protein sequence family modeling, are rarely used for modeling DNA sequence families. The algorithm commonly used to estimate the parameters of profile HMMs, Baum-Welch, is prone to prematurely converge to local optima. The DNA domain is especially problematic for the Baum-Welch algorithm, since it has only four letters as opposed to the twenty residues of the amino acid alphabet.

**Results:**

We demonstrate with a simulation study and with an application to modeling the MIR family of transposons that two recently introduced methods, Conditional Baum-Welch and Dynamic Model Surgery, achieve better estimates of the parameters of profile HMMs across a range of conditions.

**Conclusions:**

We argue that these new algorithms expand the range of potential applications of profile HMMs to many important DNA sequence family modeling problems, including that of searching for and modeling the virus-like transposons that are found in all known genomes.

## Background

Transposable elements (*transposons*) are genomic sequences that either directly encode the mechanism of their own duplication within a genome, or that appropriate a protein product from the cell or another transposable element to achieve mobility. These "jumping genes" share features and origins with viruses, though they differ from viruses in that they are usually unable to leave one cell to affect another [1]. Transposons were first characterized in 1948 by Barbara McClintock, who won a Nobel prize in 1983 for the discovery of transposons in the maize genome - of which 78% is currently identified as transposable element content [2]. Transposons have been identified in the genomes of almost all living organisms, and are especially prevalent in mammalian genomes [3]. At least 45% of the human genome is derived from transposable elements [1], as is least 38.5% of the mouse genome [4]. Another large fraction of mammalian genomes is probably transposon-derived, but has mutated to an extent that it is unidentifiable by the current approaches [3,5].

Improving transposon modeling and detection has potential impact in medicine as well as in basic molecular bioscience. Transposons are implicated in many human diseases, including hemophilia, muscular dystrophy, leukemia, breast cancer, and colon cancer [6,7]. The majority of transposons in eukaryotic genomes employ the same retrotransposing mechanisms used by retroviruses such as HIV, and many human viruses are derived from transposons (and vice-versa) [1]. Piriyapongsa and colleagues experimentally characterized 55 transposon-derived human microRNAs (miRNAs) that participate in the regulation of as many as thousands of human genes [8]. Many human genes and pseudogenes have been identified as being either derived from transposons or created by transposon activity [1]. One quarter of experimentally-identified gene promoters in mammals contain transposon-derived sequences [9]. Transposons are used to manipulate genome regulation in experimental settings and have potential applications in human gene therapy [10]. Transposon/gene location and orientation correlation suggests a positively-selected role for transposons, and has been used to enhance gene-finding algorithms [11].

This work was inspired by an earlier investigation into improving the performance of RepeatMasker (RM) [12], the popular transposon detection software, by using position-specific score matrices ("profiles", [13]). Because most transposons have been neutrally evolving for many millions of years with little selection pressure, they can be difficult to differentiate from the background distribution of genomic sequence. RepeatMasker currently uses a variant of RepBase [14], a library of consensus sequences (representing the ancestral sequences of each transposon family), and an assortment of (non-position-specific) score matrices representing different transposon ages and target sequence isochores. These score matrices are passed into the Crossmatch [15] or WU-BLAST [16-18] software packages to search a target genome for hits against each consensus sequence. The consensus-and-score matrix approach can be viewed as a simple model of the variation seen in transposon-derived sequences. Using position-specific score matrices relaxes the model's constraint that substitution patterns remain constant across the positions of the transposon. As such the use of profiles can be seen as a first step towards a more comprehensive approach to modeling the variation observed among elements of a transposon family.

The process of creating a profile or a consensus sequence from a set of sequences known to belong to a sequence family begins with the creation of a multiple alignment via insertions of gaps into each sequence so that the residues of the sequences line up. A score matrix is used to evaluate the possible ways of creating these alignments, with preference given to exact matches or chemically feasible mismatches, and to contiguous gaps. To create a consensus or profile, each column of the multiple alignment is first examined for the number or fraction of gaps, to determine if the column should be considered part of the profile (or consensus), rather than an insertion column. All of the non-insertion columns are tabulated for their content of each residue, for instance the number of adenine ("A"), cytosine ("C"), guanine ("G"), and thymine ("T") nucleotide bases. When constructing a profile, the relative frequencies of residues in a column are used to create a score matrix reflecting the column's divergence and bias (see, for instance, PSI-BLAST [19]).

The reliance on a single multiple alignment is problematic, especially since transposon sequences can be very poorly conserved. There are several popular multiple alignment programs, including ClustalW [20], T-Coffee [21], MUSCLE [22,23], and MAFFT [24]. It has been widely reported that these programs produce different multiple alignments from the same input set, and that the difference is greatest when the sequences are most diverged [25,26].

**Figure 1:**
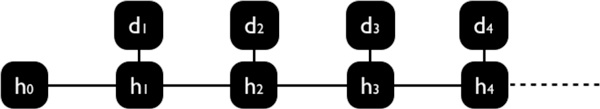
**The hidden Markov model**. The state of an unobserved Markov chain *h*_*τ *_evolves over time *τ *according to transition kernel *t*_*τ *_(·|*h*_*τ*-1_). At each time the observed datum *d*_*τ *_is distributed according to the "emission" distribution *e*_*τ *_(·|*h*_*τ*_), which depends on the current state of the hidden Markov chain.

Use of profile hidden Markov models (profile HMMs) [27,28], probabilistic models of sequence families, allows simultaneous incorporation of all of the possible multiple alignments into the determination of a profile model. The hidden Markov model (HMM), initially introduced in the late 1960s, is a powerful statistical modeling tool widely adopted in such areas as signal processing, speech recognition, and time series analysis [29]. The method was first applied to modeling biological sequences by Churchill in 1989 [30] and today is popular in biological sequence modeling [27,31,32]. HMMs assume that the distribution of an observed data point *d*_*τ *_at time *τ *∈ 1..*K *depends on an unobserved (hidden) state *h*_*τ*_. The general form of an HMM can be written as(1)

where *e*_*τ *_and *t*_*τ *_are probability distributions, and the *h*_*τ *_form a Markov chain. In this article we will follow the nomenclature of [29] and refer to *e*_*τ *_(·|*h*_*τ*_) as the "emission" distribution and to *t*_*τ *_(·|*h*_*τ *-1_) as the "transition" distribution. Figure 1 depicts the dependence structure of an HMM.

One prominent instance of HMMs in sequence analysis is the profile HMM. Profile HMMs model the residues of a genomic sequence as the observed data , with each time *τ *associated with exactly one residue *d*_*τ*_. The unobserved states of the Markov chain represent the unknown correspondence between the observed sequence's residues and the positions of the ancestral sequence from which the observed sequence has derived by a process of mutation, deletion, and insertion. The hidden data can be viewed as the alignment between the sequence and the model. The states of the Markov chain in a profile HMM correspond to ancestral positions (and to deletions of them and insertions between them).

**Figure 2:**
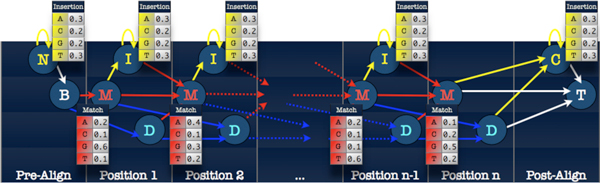
**The states of the "Plan 7" profile HMM**. There are 3*n *+ 4 states for a profile HMM representing a sequence family with *n *ancestral positions. Each internal position has three associated states: Match, Insertion, and Deletion. Additional states represent flanking insertions. All match and insertion states have an associated emission distribution, which is multinomial over the allowed residues. The insertion emission distributions typically reflect the background residue frequencies, while the match distributions are position-specific.

Figure 2 depicts the state transition diagram of the standard profile HMM model, which was explained in detail in [33] and employed in the popular software packages HMMer [34] and SAM [35]. The figure shows the "Plan 7" model used by HMMer, the name of which arises from constraints in the transition kernel disallowing all but seven transitions out of the three states corresponding to each profile position. The "Plan 9" model used by SAM is identical except that Insertion-to-Deletion and Deletion-to-Insertion transitions are also allowed.

An important feature of the HMM is the existence of a tractable algorithm, Baum-Welch, for finding maximum-likelihood and maximum a-posteriori values for its parameters [36]. The Baum-Welch algorithm is an expectation maximization (EM) algorithm for HMMs [29,37,38]. Baum-Welch iteratively improves the parameters of the distributions in Equations 1 and 2.

We recently introduced two variants of the Baum-Welch (BW) algorithm [39]: Conditional Baum-Welch (CBW) and Dynamic Model Surgery (DMS). The new variants take advantage of special constraints in the profile HMM transition kernel, and can be applied to other hidden Markov modeling contexts in which the transition kernel is similarly constrained. We showed that these variants can escape some of the local optima that infamously entrap the Baum-Welch algorithm when it is applied to profile HMMs. The phenomenon of local optima is particularly problematic in the context of DNA profile HMM models, which have fewer residues than amino acid (protein) models. While profile HMMs are widely used to model protein families, their use for DNA has been constrained by this limitation.

CBW [39] is an alternate procedure for parameterizing profile HMMs. It depends on the same update procedure as BW, but iteratively applies this procedure to conditional parameter distributions rather than to the complete joint likelihood/posterior. More precisely, CBW updates the parameters specific to a position as a group, holding all other parameters fixed, one position at a time. Non-position-specific parameters are then updated together, holding fixed the position-specific parameters, and the process is iterated until convergence. As BW is an example of the EM algorithm [37,38], CBW is an example of the Expectation Conditional Maximization (ECM) algorithm [40]. Like Baum-Welch, CBW is guaranteed to converge to a local maximum.

In the context of the profile HMM, the CBW algorithm is no less computationally efficient than the BW algorithm (indeed in practice, CBW tends to converge more quickly than BW). This efficiency is possible because the algorithm uses the same dynamic programming recursions as Baum-Welch, and most of these values do not need to be recomputed when the parameters affecting only one model position are altered.

Even with the improvements garnered by conditional maximization, the CBW algorithm often converges to local optima that are far from the global optimum. Through exploratory analysis using simulated data and profile-profile alignments, we have found that these locally optimal profile models often align with the correct profile (from which the data were simulated) after local shifts of the model positions.

We thus designed the Dynamic Model Surgery (DMS) [39] algorithm to detect misalignments and correct them. Positions that are underutilized are removed, while positions at which there are a high occurrence of insertions are duplicated. The effect is dramatic: after convergence the profiles have a much higher log-likelihood than they do without the misalignment correction.

Previously we found that under the controlled conditions of simulation studies, the new algorithms outperform Baum-Welch alone, with the best results achieved when CBW and DMS are used together [39]. In those simulation studies, summarized below, we found that for profile HMM models of DNA sequences the improvements were most dramatic when the sequences were between 60 and 70 percent conserved. Our present goal is to apply the methods to real mammalian transposon sequences of the oldest detectable age, to determine if the new approaches provide an improvement in that context. Our hypothesis is that the combination of the CBW and DMS algorithms will provide a significant improvement over BW. As described below, we find that our hypothesis appears correct for transposon models trained using transposable elements found in the human genome. As in the simulation study, we found that the new approaches perform better than the standard BW algorithm.

## Results and discussion

In [39], we demonstrated the effectiveness of the Conditional Baum-Welch (CBW) and the Dynamic Model Surgery (DMS) algorithms using simulated data. Under the controlled conditions of the simulation study it was possible to evaluate the algorithms' performance relative to the true model from which the data were generated. Here we provide a brief summary of those results, and compare them with results from a real biological data example: modeling the mammalian interspersed repeat (MIR) family of transposons in the human and mouse genomes.

### Simulation study

Our goal for the simulation study was to evaluate the relative benefits of each algorithm across a range of conservation levels. We randomly generated profile HMMs for each of the seven conservation levels in the range from .3 to .9. From each of these "true" profile HMM models we drew a set of sequences; by design the set of sequences drawn from a profile with conservation level .5 are about 50% conserved (that is, they agree with their consensus sequence at about half of their positions). We then used these training sets to estimate the parameters of a profile HMM, and compared these estimated parameters to the parameters of the "true" model. The results we provide here compare the log-likelihood of the training sequences across each combination of algorithms (Baum-Welch alone, BW with Dynamic Model Surgery, Conditional Baum-Welch alone, and CBW with DMS). We also generated another set of sequences from the same "true" profiles and used these for cross-validation. Since the algorithms are deterministic but depend on the starting values of the parameters, we used the same random "starting" profiles for every algorithm.

**Figure 3:**
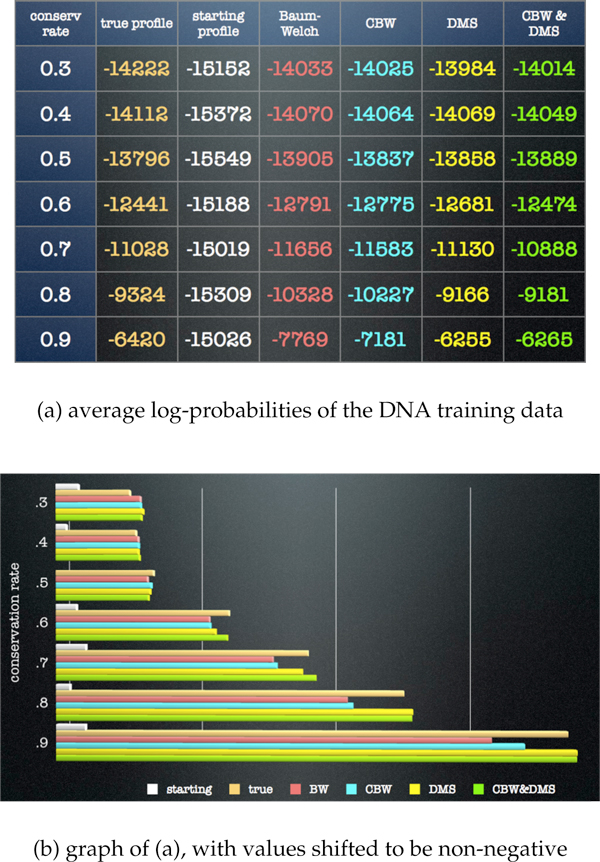
**DNA training data simulation results**. Each row in (a) and set of bars in (b) corresponds to a different conservation level in the 4 "true profiles". The first column and the top bar (white) depicts the average log-probability of the training sequences using the 16 starting profiles. The second (amber) depicts the average log-probability of the training sequences using the true profiles. The third (red) depicts the average log-probability using the Baum-Welch (BW) algorithm. The fourth (blue) depicts using the Conditional Baum-Welch (CBW) algorithm. The fifth (yellow) depicts using the Dynamic Model Surgery (DMS) algorithm with the BW algorithm, and the sixth (green) depicts using the CBW and DMS algorithms together. All bars in (b) have been shifted so that the lowest bar is at 0. The new algorithms all outperform BW at conservation levels above .6. At .5 and below, CBW does not outperform BW. At the highest levels, both algorithms employing DMS perform equally well.

**Figure 4:**
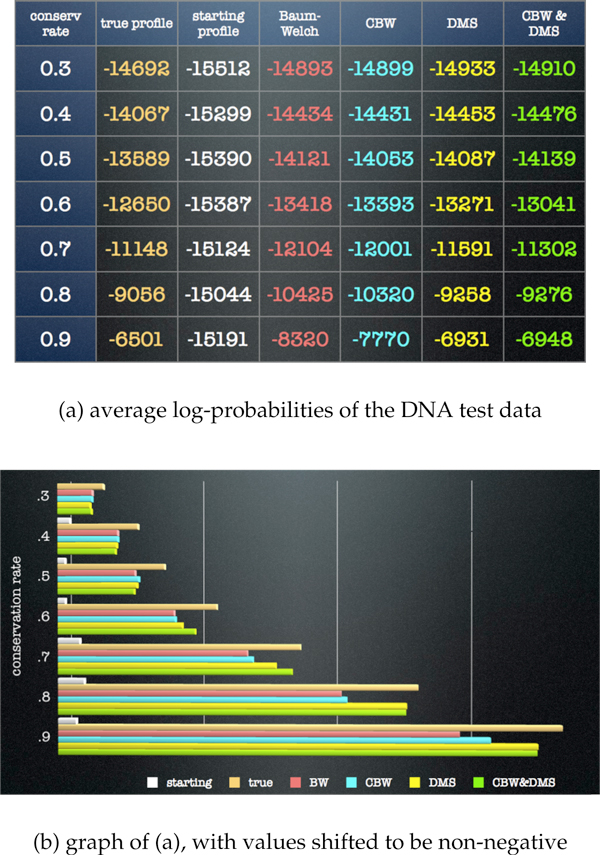
**DNA test data simulation results**. Data are represented as in Figure 3. As in the training data results in Figure 3, the new algorithms all outperform BW at conservation levels above .6. At intermediate conservation levels (.6 and .7, about the level of the oldest identifiable transposons), the algorithm employing both CBW&DMS shows the greatest improvement.

Figure 3 depicts DNA training-data results averaged over 16 runs (four starting parameter values for each of four training sets) at each conservation level. Figure 4 depicts DNA test-data results averaged over the 16 runs at each conservation level. In both the training results and the test results, the new algorithms all outperform BW at conservation levels above .5, with DMS providing a greater improvement than CBW. These results indicate that the combination of the CBW and DMS algorithms provides the greatest improvement over the other algorithm combinations at the intermediate conservation levels of .6 and .7 (about the level of the oldest identifiable transposons in the human genome). At higher conservation levels the CBW&DMS algorithms together do not outperform DMS alone, though both do better than BW or CBW alone. In an analogous simulation study for protein sequences (data not shown), the new algorithms all outperform BW at every conservation level, with DMS providing a greater improvement than CBW, and the new algorithms together demonstrating the greatest improvement.

The simulation study was designed to provide a simple setting in which to compare the methods. It did not capture the full complexity of real biological sequences. Further simulations could be performed to better reflect the natural biases in sequence residues, the transition/transversion biases in DNA substitution matrices, and other features of genomic sequences that might complicate the profile HMM parameter estimation process. Our intention is to continue exploring both simulated and real application environments for profile HMMs.

### Transitive transposons

Our goal for the transposon study was to determine if the same pattern of improvement is seen for profile HMM models of sequences generated by natural biological processes as that seen with data simulated from profile HMMs. Real genomic sequences are generated by a complex set of biochemical processes that are never fully representable in a mathematical model, and it is possible that our simulation results fail to capture an important feature of real genomic data.

We aim to build profile HMM models to identify transopon elements in the mouse and human genomes. Due to the shorter generation time in mice, transposons are on average 1.7 times more diverged in the mouse genome than in the human genome, and are thus more difficult to identify [1]. Comparative genomics can be used to identify transposons in one genome by homology with those found in another genome. Using profile HMMs, we hope to identify transposon elements in the mouse and human genomes that are not identified by RepeatMasker. We then use genome-genome homology with known elements in the other genome to confirm our findings.

The mammalian interspersed repeat (MIR) family, at between 60 and 70 percent conserved, is one of the most diverged transposable element families identifiable in the human genome by current techniques. We put together a training set of about 200 sequences of MIR elements found in the human genome by RepeatMasker. We used these sequences to train profile HMMs (using each combination of algorithms) from a variety of random start profiles, and then evaluated the methods by searching for new elements in target regions of the mouse and human genomes known to be mutually homologous. We restricted the size of the training set to more easily compare the methods, though in practice we will use all available data when building models for deployment.

In addition to calculating the number of elements found by each profile HMM training procedure that are homologous to known elements on the other genome, we also evaluated the procedures using a measure of the quality of the alignments. In particular we are interested in the quality of what we deem the "transitive alignments," the alignments between the known elements on one genome and the newly found elements on the other genome. We measured quality by the number of exact matches in each transitive alignment, plus the number of transitions (which are the more chemically favorable mutations within purine (A, G) and pyrimidine (C, T) nucleotide sets, as opposed to transversions, which are the other, less probable mutations).

**Figure 5:**
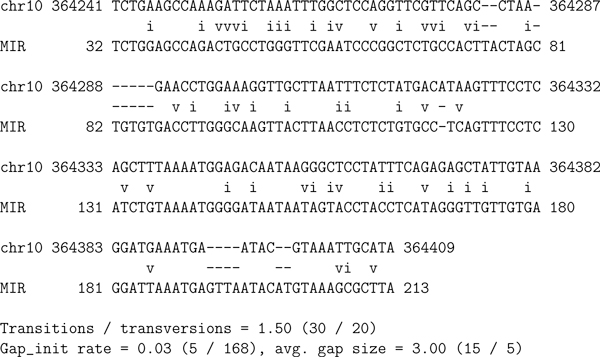
**RepeatMasker MIR transposon element at hg18 chr10:364, 241-364, 409**. Pairwise alignment created by RepeatMasker [12] using Crossmatch [15]. The top line of each block is the segment of human genome (version 18) found to be a "hit" to the MIR transposon family consensus sequence, shown on the bottom line of each block. The middle line shows exact matches (spaces), gaps (dashes) and mismatches ("i" for transitions and "v" for transversions). This is an element for which a mouse analogue was not found by RepeatMasker, but was known to exist by human-mouse homology. The mouse analogue was found using a profile HMM.

To illustrate how the transitive alignment metric works, consider the human genome MIR element at hg18 chr10:364, 241-364,409. Figure 5 depicts the pairwise alignment between that element and the human genome region on which it is found.

**Figure 6:**
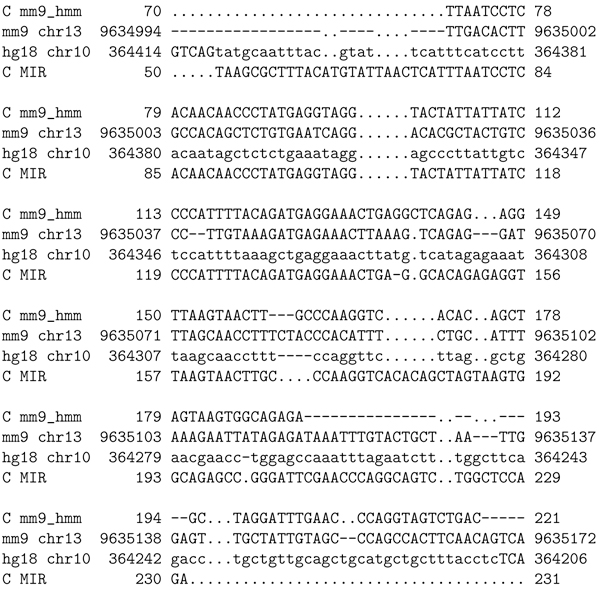
**Transitive pileup multiple alignment depicting the new element in the context of the mouse-human homology, with the human MIR element found by RepeatMasker**. The first line depicts the complemented MIRc element found via profile HMM on chromosome 13 of the mouse genome. The second line is the corresponding region of the mouse genome. The second and third lines together depict the relevant fragment of the mouse-human alignment (from UCSC's chain/net alignments). The bottom line is the MIR hit found by RM on hg18. This is an excerpt; the mm9 element extends further on both ends.

The region of the mouse genome corresponding to the MIR element, according to the UCSC genome-genome alignment, is the complement strand of hg18 chr13:9,634, 994-9, 635, 139. One side of the RM alignment extends beyond the genome-genome alignment. The multiple alignment in Figure 6 shows a new element on the mouse genome that we found using a profile HMM, in the context of the mouse-human homology.

**Figure 7:**
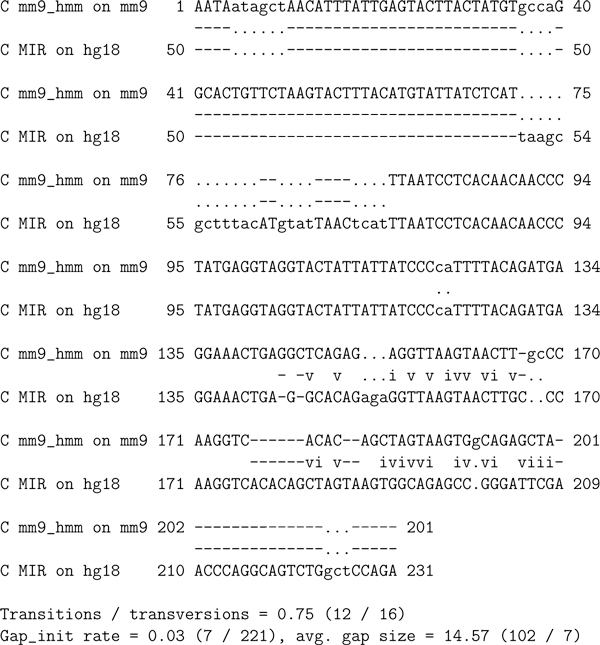
**Transitive alignment comparing the new MIR element found in mouse using a profile HMM and the MIR element found in human by RepeatMasker**. Generated by removing the genome lines from the transitive pileup multiple alignment depicted in Figure 6, and adding the usual pairwise lines depicting matches, gaps, transitions, and transversions. Lowercase characters represent regions of one element that could not possibly align transitively because the genome-genome alignment contains a gap there. Gap symbols at such positions are represented as dots "." rather than as dashes "-". Note that the statistics at the bottom are computed after removing all such positions of the alignment. The matches and transitions from transitive alignments are used to compare the profile HMM parameterization algorithms.

From this transitive pileup multiple alignment, pairwise alignments between the new "hit" (the putative new element) on the mouse genome and the known element on the human genome are implied (by removing the genome lines). These are what we refer to as "transitive alignments." The transitive alignment between the new element and the human MIR element is depicted in Figure 7.

Transitive alignments contain many more gaps than do non-transitive alignments. The majority of the gaps are flanking gaps; internally, these alignments are quite good. We have found that the boundaries between the flanking gaps and the internal alignments are usually defined by dot-gaps, indicating that there is a gap in one of the genomes relative to the other. These genome alterations can force one hit to terminate while the other continues.

Our comparison metric is the number of matches in the transitive alignments between the hits we find on one genome and the RM hits on the other genome, plus the number of transitions. For instance in the transitive alignment depicted in Figure 7 there are 91 matches and 12 transitions (plus 16 transversions and 102 gaps). In the 550 fragments of homology between mm9 chr13:9,000,000-10,000,000 and hg18 chr10:1-1,000,000, RepeatMasker finds 28 MIR elements (15 in human, 13 in mouse), of which 8 (4 in each genome) transitively align. These 4 RM-RM transitive alignments have 394 matches, plus 27 transitions, for a total of 421.

**Figure 8:**
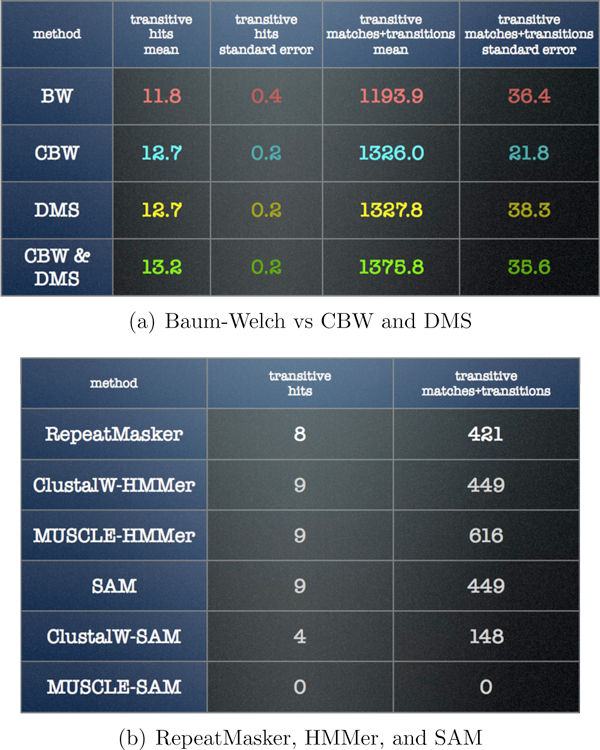
**Transposon data results**. The table in (a) shows the average, over 16 runs from different starting profiles, of the number of transitive hits and the total number of matches and transitions in those transitive alignments. All three new algorithm variants show significant improvements over Baum-Welch, and the CBW algorithm exhibits the greatest improvement. The table in (b) shows the RepeatMasker results, results using HMMer-2 in conjunction with ClustalW and MUSCLE, and results using SAM with unaligned inputs and with aligned inputs.

Figure 8 depicts a summary of the results of applying the Baum-Welch method and its new variants to the transposon search problem. For comparison we also provide the results of using the popular HMMer and SAM profile HMM software packages. The current version of HMMer does not apply the Baum-Welch algorithm, and SAM (which is closed-source) employs variants that are unknown to us at this time, but which clearly did not perform as well as even Baum-Welch for this problem.

All three new algorithm variants show significant improvements over Baum-Welch. The same pattern of improvement is seen as in the simulation study: CBW with DMS is the best combination, CBW alone provides an improvement over BW alone, and the BW & DMS combination provides more improvement over BW than does CBW alone. In this case the differences among the three new combinations is only slight, and is within the margin of error.

The high variability of the Baum-Welch results reflects the higher tendency of that algorithm to get stuck in local optima. The DMS algorithm was more difficult to apply to this context than to the simulated data because these data show many more deletions than insertions. To accommodate this, we had to adjust a tuning parameter that governs the threshold at which positions are inserted or removed. While we put some effort into finding the parameter value that optimized the model length, the resulting models had higher length variability than models in our simulation study. The HMMer and SAM packages required tuning of analogous parameters. Details are provided in the Methods section.

The comparison to RepeatMasker and to existing profile HMM packages indicates the promise of the new approaches. The MUSCLE-HMMer combination seems to improve upon RepeatMasker, but the number and quality of transitive alignments for all of the methods in Figure 8(a) are much higher than those in Figure 8(b).

## Conclusions

Profile HMMs are a model-based alternative to the ad-hoc multiple alignment approaches common for sequence family modeling. Since different multiple alignment software packages can yield very different alignments, use of the profile HMM approach is more robust than relying on a single multiple alignment to construct a consensus or a position-specific score matrix.

While profile HMMs are widely used for protein family modeling, and databases of profile HMMs exist for protein sequence families, including the popular Pfam [41] and SUPERFAMILY [42] databases, profile HMMs have not generally been embraced as a tool for modeling DNA sequence families. Both of the major software packages for building and using profile HMMs explicitly focus on protein family modeling, though they retain some support for DNA. The Baum-Welch algorithm, which has problems with local optima in the context of protein sequences, is even more prone to premature convergence when there are four residues instead of twenty. Our hope is that the algorithmic contributions described here and in [39] will open new possibilities for applications of profile HMMs to the DNA domain.

The simulation study and transposon study both demonstrate that the Conditional Baum-Welch algorithm is an improvement over the Baum-Welch algorithm, as is the Dynamic Model Surgery algorithm. The simulation study demonstrated a greater improvement from the DMS algorithm than from the CBW algorithm, with the best improvement when the algorithms are used together. In the transposon study, each of the new algorithm combinations provide a large improvement over BW alone, but we found little discernible difference among the new approaches. The DMS algorithm always converged on a model length near the RepeatMasker consensus length, though it required tuning of a threshold parameter to do so. The HMMer and SAM packages also require parameters to be tuned to control the model length, and these packages performed relatively poorly even after tuning.

We have argued that improving transposon modeling is a worthwhile endeavor, and have shown that, by one metric, the new methods do improve upon the transposon-finding abilities of existing profile HMM parameterization methods. The new algorithms overcome one of the major impediments to the application of profile HMMs in the transposon modeling problem: that of poor parameter estimation. Already new repeat elements have been identified using these new techniques, and we will work with the transposon research community to develop the methods further.

## Methods

### Baum-Welch

BW [37] iteratively improves the parameters of the distributions in Equations 1 and 2. The utility of the algorithm relies on an efficient recursive formula for calculating the "forward" values *α*_*τ *_(*h*_*τ*_) = ℙ(*d*^*τ*^, *h*_*τ*_|**Θ**), where the notation *d*^*τ *^denotes the first *τ *elements (e.g. residues) of observed sequence , and **Θ **are the parameters of the HMM.

The probability of the sequence  given the parameterized model is easily calculated from the forward value, since(3)

where  is the state space of the Markov chain, and *K *= || is the number of observations in .

The dynamic programming recursion for computing *α*_*τ *_as a function of *α*_*τ*-1 _follows from the conditional independence assumptions of the Markov chain underlying the HMM:(4)

The "backward" value *β*_*τ*_(*h*_*τ*_) = ℙ(*d*^-*τ*^|*h*_*τ*_, **Θ**) is the conditional distribution of the remaining *K *- *τ *components of  (after *d*_*τ*_), which we denote by *d*^-*τ*^, given the state *h*_*τ *_at time *τ*. The recursion for calculating the backward value is similar to that of the forward value, only in reverse:(5)

Alone, either the forward or the backward values can be used to efficiently calculate the likelihood function (Equation 3). Together, the forward and backward values yield the conditional distribution of the hidden state *h*_*τ *_given the observed sequence , since

With uninformative (e.g. Laplace) priors on the parameters, this can be interpreted as the posterior probability that the HMM emitted the *τ*^*th *^element of sequence  from state *h*_*τ *_at time *τ*. More complex priors are also possible via the usual procedure, with conjugate (e.g. Dirichlet) priors preferable for computational simplicity.

In each iteration, the BW procedure updates the parameters of the "emission" distributions *e *and the parameters of the "transition" distributions *t *such that each parameter value is proportional to the average (over all of the observed sequences) of the expected number of uses of the corresponding emission (or transition). For example, the update to the probability of emitting datum *d *at state *h ∈ * is

where **1**(·) is the indicator function and where *d*_*τ *_is the *τ*^*th *^residue in observed sequence  (and we sum over all of the observed sequences {}).

Similarly, the BW update for the probability of transitioning from some state *h*_*A *_to another state *h*_*B *_is

The BW algorithm iterates between calculating the forward-backward values and updating the parameters, until convergence. The convergence point depends deterministically on the data, and unfortunately also on the starting parameters. In the case of profile HMMs, the convergence point is highly sensitive to the starting parameters.

### Conditional Baum-Welch

The CBW algorithm updates subsets of the parameters as a group, conditional on the other parameters. Applied to the profile HMM, for example, the CBW algorithm separately updates the transition parameters and Match emission parameters associated with each model position (cf. Figure 2). CBW updates groups of state-dependent parameters together, holding fixed the values of the other parameters. State-independent parameters (such as the parameters of the Insertion emission distribution of the profile HMM) are then separately updated together, holding fixed the values of the other groups of parameters.

For CBW to be as efficient as BW, the transition probability matrix of the underlying Markov chain must be relatively sparse and relatively diagonal. In particular, there must exist an ordering of the states  = *s*_1_,..., *s*_*S *_such that the probability *t*(*s*_*i*_,*s*_*j*_) of transitioning from state *i *to state *j *is zero unless *j ≥ i *and (*j - i*) ≤ *k *for some fixed small constant *k*. For the Plan 7 and Plan 9 profile HMM models, the condition is satisfied with *k *= 5 (from a Match state at one position to the Deletion state at the subsequent position).

When this condition is satisfied, the forward and backward dynamic programming recursions can proceed state-by-state rather than time-by-time. That is, instead of computing the forward values *α*_*τ*_(*h*_*τ*_) for time *τ *as a function of the forward values for all states at the previous time (as in Equation 4), the values can be computed for state *s *as a function of the *k *previous states at the previous time, since(6)

The CBW algorithm computes the forward and backward values row-by-row (state-by-state) rather than column-by-column (time-by-time). Since the calculation of the forward values in a row depends only on the parameters affecting the states of that row, and on the forward values of the previous row, the CBW algorithm only needs to recompute the forward values of the affected row. Since each forward value needs to be calculated only once, the total computational cost of the CBW algorithm is on the same order as that of the BW algorithm (bilinear in the number of states and the total length of all observation sequences).

### Dynamic Model Surgery

DMS makes use of the same row-by-row parameter update computations that are used by CBW. While CBW uses these calculations to update the parameter values of a profile HMM, DMS uses them to dynamically change the model's structure. The DMS algorithm can also be used in conjunction with the BW algorithm, so long as BW is implemented as a "delayed" CBW, as described in [39].

The DMS algorithm identifies positions at which there are an excessive number of insertions or deletions. Positions that are underutilized are removed, while positions at which there is a high occurrence of insertions are duplicated. The effect is dramatic: after convergence the profiles have a much higher log-likelihood than they do without the misalignment correction. We have shown in [39] that under a broad range of simulation conditions the DMS algorithm, used in conjunction with either BW or CBW, outperforms both BW and CBW alone.

Altering the model structure helps the parameter estimation algorithms escape from local optima caused by poor use of the model's positions. To understand this it is helpful to think of the positions of the profile HMM as corresponding to columns of a multiple alignment. The problem is analogous to having some of the alignment columns contain mostly gaps: these should be identified as "insertion columns"; in the profile HMM context, there should not be a match state associated with such columns (there should be an insertion state instead). Another problem is model positions that are overutilized: they represent more than one column of the multiple alignment, and since each position has only one match state, all emitted residues after the first are misrepresented as  insertions. This misalignment problem can be corrected by adding and removing model positions.

DMS is inspired by the Model Surgery technique introduced in [27] and implemented in SAM [35]. Both Model Surgery and DMS alter the structure of the profile HMM during training by analysing the usage of the model's states. SAM's Model Surgery algorithm applies these structural changes as a group after BW converges, then reruns BW. DMS is a dynamic alternative, in that its structural changes are applied throughout the parameter estimation process.

The DMS algorithm uses the BW or CBW update calculations to determine if a model position is underused (in which case, it removes that position) or if a model position is overused (in which case a new position is introduced into the model). In particular, the expected counts for transitions into the Insertion and Deletion states are compared to a threshold *ξ*. If more than some fraction *ν *(e.g. 50%) of the sequences have expected insertion counts exceeding *ξ *at a position *j*, then an additional position is added to the model after position *j*. If more than 1 - *ν *of the sequences have expected deletion counts exceeding *ξ*, then the position is removed from the model.

The DMS algorithm requires computing, for each position *j*, the fraction  of sequences for which the expected insertion count at position *j *exceeds the threshold *ξ*, and the fraction  of sequences for which the expected deletion count at that position exceeds *ξ*.  and  are computed while the BW or CBW update for each position is being calculated.

The efficiency of the row-orientated update procedure makes it possible to incorporate structural changes immediately. The row-wise orientation of the CBW forward-backward procedure ensures that the dynamic programming calculations do not need to be recomputed except at the affected states. Backward values for subsequent rows are not affected by the change, since the backward recursion concerns only the subsequent states. Likewise, forward values for preceding rows are not affected, since they depend only on the states preceding them, which are unchanged.

### Simulation study

For the simulation study, we generated sets of sequences from profile HMMs with known parameters. We split each set of generated sequences into a training set and a test set, and then assessed how well the algorithms performed on the test set after being parameterized with the training set. The profile HMMs from which the training and test sequence sets were drawn were generated by first drawing one random 100-length "true consensus" sequence for each. We then set the multinomial emission distribution at each position of the "true" profile to assign more probability to that true consensus residue than to the others. For instance if the randomly drawn true consensus residue at a position was "A" and the conservation level was .5, then we assigned .5 probability to "A" at that position. For simplicity, we evenly divided the remaining (1.0 - .5) probability among the other residues. Each profile was then used to generate 100 training sequences and 100 test sequences. We also created four uniformly-distributed starting profiles for each true profile. We separately ran each algorithm on the 100 training sequences from every starting position.

The algorithms employing Dynamic Model Surgery were set to insert a profile position whenever the number of sequences with insertion fractions exceeding the DMS threshold was at least 50% of all training sequences, and to delete a position whenever the number of sequences with deletion fractions exceeding the DMS threshold was at least 50% of all training sequences (that is, with *ν *set to .5). The DMS threshold *ξ *was set to begin at .01 and to increase by ϵ = .005 whenever a cycle was detected. The algorithms were trained until convergence, with convergence defined as the average euclidean distance of all free parameters being less than 10^-5^. For additional details, as well as a discussion of the limitations of the simulation study, we refer the interested reader to [39].

### Transposon study

To summarize, we built profile HMM models from known elements of the MIR transposon, and compared out-of-sample hits found using these models to known elements found by RepeatMasker. We retrieved FastA sequences of MIR elements from the RepeatMasker web service [12] for the human (hg18) genome. We filtered these elements based on repeat length, and then used these filtered repeats to train separate profile HMMs for each method. We created 18 randomly-determined starting parameter values per genome. For each random start profile, we applied each algorithm (BW, CBW, DMS&BW, DMS&CBW), until convergence. The resulting profile HMMs were used to search for hits in a region of the human genome not used for training and in a region of the mouse genome homologous to the human region. These models were also used to search for hits in randomly shuffled variants of the true target regions. For each profile, we ordered the scores of the hits from the shuffled sequence to establish a threshold above which only 5% of those hits fell, to be used as the minimum score threshold for hits from the corresponding true (not shuffled) target region.

These hits were used to create transitive alignments using the genome-genome alignment of mouse and human (from the net/chain database at UCSC) and the MIR hits found by RepeatMasker in the target region. We then transitively aligned mouse profile HMM hits to human RM hits and vice-versa. For each transitive alignment, we computed the number of matches and transitions that are shown in Figure 8(a).

We now provide specifics of how the transitive transposon results were generated. The profile HMMs were trained using the 206 MIR elements found by RepeatMasker in human (hg18) chromosome 22 that are least 150 residues in length. The region on the human genome used to test the trained profiles was hg18 chr10:1-1,000,000. According to the chain/net genome-genome alignments from UCSC, the region has 550 fragments aligning to mm9 chr13:9,000,000-10,000,000. For this reason we used mm9 chr13:9,000,000-10,000,000 as the mouse genome test region. RepeatMasker identifies 15 MIR elements in the human genome that overlap with these chain/net fragments, and 13 MIR elements in the mouse genome that overlap with these fragments. 8 of these MIRs, 4 on each genome, transitively align via the fragments.

The profile HMMs were trained from 18 different starting profiles per method. The profiles were of length 262 (the length of the RepeatMasker MIR consensus sequence) for the BW and CBW methods. For the methods employing DMS, the length of the starting profiles was set to the median length of the training sequences, which was 173. The Match emission distributions of these profiles were randomly drawn from Laplace distributions. The Insertion emission distributions were even (.25 on each residue). The Insertion and Deletion state transition probabilities were even (.5 to extend the gap, .5 to end the gap). The profile HMM algorithms were all set to "local" mode, meaning that flanking insertions were disallowed and extra deletion-in and deletion-out transitions were possible. The transitions from the Match state were set with probabilities .9 to enter the DeletionOut state, .095 to transition to the subsequent Match state, .0025 to transition to the Insertion state, and .0025 to transition to the subsequent Deletion state. The transitions from the Begin state were set with probabilities .9 to enter the DeletionIn state, .09 to enter the first Match state, and .01 to enter the first Deletion state. The transition probabilities from the DeletionIn and DeletionOut states were set such that continuing the deletion had probability .9999, and ending it had probability .0001. All of these parameters were subject to alteration by the algorithms, except for the flanking insertion probabilities, which were fixed to disallow flanking insertions.

The average length of the profile HMMs that were trained using DMS was 259.5 for DMS with BW, with a standard deviation of .9, and 259.1 for DMS with CBW, with a standard deviation of 1.0. The RepeatMasker consensus sequence for this transposon has length 262. The lengths after convergence are strongly influenced by a parameter, essentially equivalent to the "gapmax" parameter of HMMer or the "mainline_cutoff" parameter of SAM. We tuned this parameter to get model lengths reasonably close to the consensus length. As described below, we did the same for the HMMer and SAM models. Note that with these sequences the DMS algorithm exhibits some variation in the model length (in contrast to the simulation study, in which the lengths were quite accurate). The DMS length results are very resilient to starting profile length, with no notable difference between the length distribution of models starting from length 262 (not shown) and that of models starting from the median length of the training sequences (as shown here).

Weak Dirichlet priors were used in all algorithms for all of the parameters. In the case of the Match emission distributions, the priors were set to Laplace distributions. Priors for the other parameters were set with the same parameters as the starting values described above, with the Match, Insertion, Deletion, DeletionIn and DeletionOut state transition priors scaled by the initial profile length, to reflect their multiple use. In addition to the priors, a minimum value of 10^-5 ^was enforced for all parameter values being trained, to prevent the algorithms from getting trapped with zero-valued parameters.

The algorithms employing dynamic model surgery (DMS) were set to insert a profile position whenever the number of sequences with insertion fractions exceeding the DMS threshold was at least 10% of all training sequences, and to delete a position whenever the number of sequences with deletion fractions exceeding the DMS threshold was at least 90% of all training sequences. This roughly reflects the heuristic used by the authors of RepeatMasker, who include a residue in a transposon's consensus sequence whenever the multiple alignment column contains at least 4 non-gaps. The DMS threshold was set to begin at .1 and to increase whenever a cycle was detected to the minimum amount that would change the fraction of sequences exceeding it.

The algorithms were trained until convergence, with convergence defined as the average euclidean distance of all free parameters being less than 10^-7^.

After training, the resulting profile HMMs were converted to HMMer-2 format. This conversion procedure effectively prepares the HMMer-style HMM for "FS" style search (local in both sequence and model, with the possibility of finding multiple instances of the model in the sequence). We used the profile HMM's insertion distribution for the null model emission distribution, and .25 for the null model termination probability and for the loop-state (inter-hit insertion) termination probability. These values imply an expectation of only 4 insertions between hits, which is lower than we actually expect. All other model probabilities are transferred unchanged into HMMer-2's HMM format.

The HMMer-2 hmmsearch program was used to search each of these converted HMM files against the relevant test region (mm9 chr13:9,000,000-10,000,000 or hg18 chr10:1-1,000,000). No arguments were given to this program except for the HMM file and the sequence FastA file. The resulting hits that exceeded the predetermined threshold were then converted to cross-match-style pairwise alignments, then transitively aligned to the RepeatMasker hits on the other genome. The transitive alignments were counted and the matching residues tallied.

### HMMer and SAM

For comparison we also ran the popular profile HMM software packages HMMer and SAM on the same training data sets, and computed transitive alignments exactly as described above. The documentation for HMMer-2 warns against its use for DNA sequence family modeling, and indeed the default options created tiny (length 1) profiles. Regardless of the options, HMMer-2 does not actually implement Baum-Welch, and so it requires pre-aligned input sequences. We ran ClustalW [20] using the options "-align -type=dna -dnamatrix=clustalw -pwdnamatrix=clustalw -outorder=input" and MUSCLE [22,23] using the options "-stable -maxiters 3". We used these to build profile models using the hmmbuild program of HMMer-2 (version 2.3.2) with the options "-f -nucleic -fast -gapmax 0.9 -wnone". We tried it without the -fast option, but the resulting profiles had length 1, as mentioned above. We experimented with the -gapmax option, and found that .9 gave reasonable-length models (model lengths were 338 with MUSCLE, 279 with ClustalW).

The SAM program no longer supports DNA sequence family modeling through the web interface, but version 3.5 of the SAM buildmodel program allows it with the "-alphabet DNA" option. We also supplied the "-mainline_cutoff" option, which controls SAM's variant of model surgery and thus effectively controls the profile length. We searched for the optimal value of this option separately for each profile that we built with SAM, among multiples of .05. Since SAM accepts both aligned and unaligned sequence inputs, we build a total of three SAM models: one from the unaligned sequences, and one each from the ClustalW and MUSCLE alignments used for the HMMer profile building process. The resulting profile lengths were 249 for MUSCLE alone, 240 with ClustalW, and 255 with MUSCLE. We converted the resulting SAM profiles to HMMer-2 format and calculated transitive alignments, as described above.

## Competing interests

The authors declare that they have no competing interests.

## Authors' contributions

PE developed the methods, programmed the software, carried out the simulation and transposon studies, and drafted the manuscript. JL contributed to the development of the methods and helped to draft the manuscript.
